# Mesenchymal Stem Cells in Renal Fibrosis: The Flame of Cytotherapy

**DOI:** 10.1155/2019/8387350

**Published:** 2019-01-13

**Authors:** Quan Zhuang, Ruoyu Ma, Yanshuang Yin, Tianhao Lan, Meng Yu, Yingzi Ming

**Affiliations:** ^1^Transplantation Center of The 3rd Xiangya Hospital, Central South University, Changsha, Hunan 410013, China; ^2^Research Center of National Health Ministry on Transplantation Medicine, Changsha, Hunan 410013, China; ^3^Xiangya School of Medicine, Central South University, Changsha, Hunan 410013, China

## Abstract

Renal fibrosis, as the fundamental pathological process of chronic kidney disease (CKD), is a pathologic extension of the normal wound healing process characterized by endothelium injury, myofibroblast activation, macrophage migration, inflammatory signaling stimulation, matrix deposition, and remodelling. Yet, the current method of treating renal fibrosis is fairly limited, including angiotensin-converting enzyme inhibition, angiotensin receptor blockade, optimal blood pressure control, and sodium bicarbonate for metabolic acidosis. MSCs are pluripotent adult stem cells that can differentiate into various types of tissue lineages, such as the cartilage (chondrocytes), bone (osteoblasts), fat (adipocytes), and muscle (myocytes). Because of their many advantages like ubiquitous sources, convenient procurement and collection, low immunogenicity, and low adverse effects, with their special identification markers, mesenchymal stem MSC-based therapy is getting more and more attention. Based on the mechanism of renal fibrosis, MSCs mostly participate throughout the renal fibrotic process. According to the latest and overall literature reviews, we aim to elucidate the antifibrotic mechanisms and effects of diverse sources of MSCs on renal fibrosis, assess their efficacy and safety in preliminarily clinical application, answer the controversial questions, and provide novel ideas into the MSC cellular therapy of renal fibrosis.

## 1. Introduction

Renal fibrosis develops following an accumulation of scar tissue within the parenchyma, and it represents the collaborative ultimate pathway of nearly all the chronic and progressive nephropathies [1]. Affecting more than 10% of the world population with limited treatment options, renal fibrosis remains a major public health conundrum as it is considered the fundamental pathological process of chronic kidney disease (CKD) independent of the underlying etiology [2]. CKD is also one of the strongest risk factors for cardiovascular disease [3, 4]. Although the idea of reversing CKD has been investigated by scientists repeatedly in the past decade, existing treatments that prevent CKD progression and CKD-related complications are quite limited [5] and currently include angiotensin-converting enzyme inhibition, angiotensin receptor blockade, optimal blood pressure control, and sodium bicarbonate for metabolic acidosis [6]. Therefore, the prevention or reversal of renal fibrosis remains ineffective or only slightly successful, and the development of a new strategy for the treatment of this pathological process is extremely urgent.

**Figure 1:**
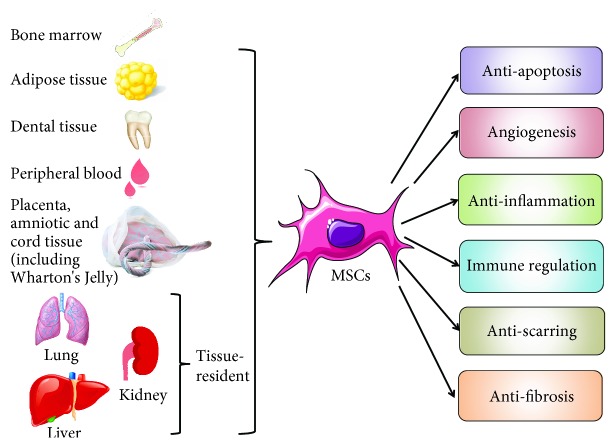
Different sources and types of MSCs and their function in different pathophysiological processes.

To date, an increasing number of studies have shown that stem cell treatment is prominently effective in chronic and progressive diseases [7]. Multiple types of stem cells, including mesenchymal stem cells (MSCs) [8], embryonic stem cells (ESCs) [9], and induced pluripotent stem cells (iPSCs) [10], have manifested their qualities as viable and accessible sources for tissue repair and regeneration. Because of the ethical and expense issues, MSCs exhibit advantages compared to ESCs and iPSCs [11]. MSCs are pluripotent adult stem cells that can differentiate into various types of tissue lineages, such as the cartilage (chondrocytes), bone (osteoblasts), fat (adipocytes), and muscle (myocytes) [12]. The International Cell Therapy Association has established the minimum standard for human MSC definition [13]: cells must be plastically adherent; exhibit a three-lineage differentiation in osteoblasts, adipocytes, and chondrocytes; and express certain surface patterns of CD105, CD73, and CD90, while lacking CD45, CD34, CD14, CD11b, or CD79a or the expression of CD19, as well as HLA-DR. Moreover, Gli1 may be used as a marker for MSCs according to recent research reports [14]. Since then, MSCs have been proven to be derived from virtually all tissues' adventitial progenitor cells and pericytes [15]. The most applied tissues include the bone marrow [16], cord cells [17], adipose tissue [18], molar cells [19], amniotic fluid [20], and placenta [21], as well as several solid organs, such as the lung [22], liver [23], and kidney [24]. These MSCs from solid organs are referred to as tissue-resident MSCs [25]. Since Friedenstein and Caplan first defined MSCs according to their multilineage potential [26, 27], MSCs have shown their cellular therapeutic competence in many diseases and pathopoiesis. It is widely agreed that transplanted MSCs can directly reconstruct impaired organs [28]. MSCs are also capable of producing cytokines, growth factors, and chemokines; moreover, they exert a comprehensive range of functions by expressing extracellular matrix receptors on their cell surface, including antiapoptosis [29], angiogenesis [30], anti-inflammation [31], immune regulation [32], antiscarring [33], and chemically induced homing to damaged tissue, thus supporting the growth and differentiation of diseased cells, which makes them attractive for clinical applications. Fibrosis, as one of the most common and refractory pathological processes, has always drawn substantial attention, and many efforts and trials of MSC cellular therapy have been carried out on antifibrotic diseases [34]. Pondering the origin and therapeutic activities of MSCs, we summarize this network in Figure 1.

In this review, we will discuss the antifibrotic mechanisms and effects of different sources of MSCs on renal fibrosis and evaluate their efficacy and safety in preliminarily clinical application, aiming to provide overall and new insights on MSC cell therapy in renal fibrosis.

## 2. The Link between Renal Fibrosis and MSCs

Renal fibrosis features a redundant accumulation of extracellular matrix (ECM), which undermines and supplants the functional parenchyma that results in organ failure. Consequently, the imbalance between a superfluous production and lessened reduction of the ECM results in tubulointerstitial fibrosis and glomerulosclerosis. Glomerulosclerosis may be elicited by metabolic, mechanical, or immunological impairments on endothelial cells or podocytes, resulting in an increasing production of ECM by mesangial cells [35]. However, it is commonly agreed that tubulointerstitial fibrosis plays the main role by depositing the matrix protein between the basement membrane and peritubular capillaries.

Four stages may be observed during the pathophysiology of renal fibrosis: (1) cellular activation and injury phase or priming, (2) fibrogenic signaling stimulation phase, (3) fibrogenic phase or execution, and (4) destructive phase or progression [36]. During these phases, several signaling pathways are strongly involved, including nuclear factor-kappaB (NF-*κ*B) [37], transforming growth factor-*β*1 (TGF-*β*1)/small mothers against decapentaplegic (Smad) [38], Notch, wingless-type MMTV integration site (Wnt), Hedgehog [39], protein kinase C (PKC)/extracellular regulated protein kinases (ERK), and phosphatidylinositol-3 kinase (PI3K)/Akt [40]. However, TGF-*β*1/Smad signaling has been considered the central pathway that mediates renal fibrosis and CKD progression among these signaling pathways because TGF-*β*1/Smad has ubiquitous communications with other signaling pathways in the fibrotic process [41]. The potential mechanism of renal fibrosis and CKD has been described as follows based on the 4 phases previously noted. Renal epithelial cell injury may be elicited by ischemia, toxins, and proteinuria from many diseases, such as glomerulonephritis, diabetes, or hypertension, and it may lead to fibroblast proliferation [42] and macrophage infiltration [43]. TGF-*β*1 are consequently released from the damaged and infiltrating cells and act on fibroblast-type cells in the kidney (that is, mesangial cells and fibroblasts) [44], which subsequently induce the epithelial-to-mesenchymal transition (EMT) and cause more ECM deposition [41]. Finally, profibrotic molecules, including collagens, fibronectin, and plasminogen activator inhibitor-1 (PAI-1), may increase and lead to kidney failure [44].

**Figure 2:**
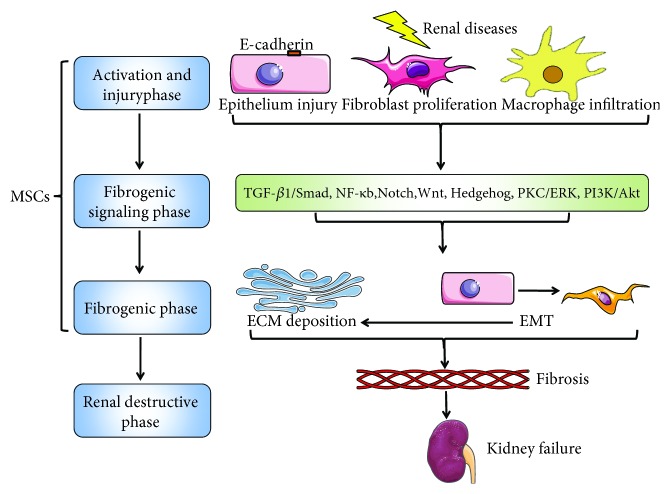
The link between renal fibrosis and MSCs. MSCs, as the protectively fibrotic mediators, play the crucial role in the former 3 phases during the renal fibrotic process. Phase 1: activation and injury phase. Renal diseases lead to epithelial cell injury (E-cadherin shedding), fibroblast proliferation, and macrophage infiltration. Phase 2: fibrogenic signaling phase. Inflammatory and fibrotic signaling pathways are activated. Phase 3: fibrogenic phase. EMT increase and ECM deposition (EMT also could cause ECM deposition). Phase 4: destructive phase. Fibronectin, collagens, *α*-SMA increase, and finally renal failure.

MSCs, as protective fibrotic mediators, play the crucial role in at least the former 3 phases during the renal fibrotic process (Figure 2). Bone marrow-derived MSCs (BM-MSCs) could affect the cellular activation and injury phase through inflammation by reducing the expression of proinflammatory cytokines [45], and it reduces the fibrogenic signaling stimulation phase by signaling pathways, mainly TGF-*β*1/Smad, as well as NK-*κ*B and ERK. BM-MSCs can inhibit the fibrogenic phase or execution by restraining the EMT [46]. Umbilical cord-derived MSCs (UC-MSCs) can affect the fibrogenic signaling stimulation phase, such as TGF-*β*1 [47], TLR4/NF-*κ*B [48], ERK [41], and Akt [49] signaling pathways. They can also reduce EMT. Additionally, adipose-derived MSCs (AMSCs) can participate in the cellular activation and injury phase or priming by reducing the release of IL-1*β*, TNF-*α*, and IL-6. AMSCs could also inhibit TGF-*β*1/Smad2/3/7 signaling pathway activation [50]. Therefore, we will discuss the antifibrotic mechanisms and effects of different sources of MSCs on the former 3 fibrotic phases as follows.

## 3. Mechanisms and Functions of MSCs in Renal Fibrosis Therapy

### 3.1. Bone Marrow-Derived MSCs

BM-MSCs were the first identified MSCs by Friedenstein et al. in 1966 [26]. Various animal studies have corroborated that extraneous BM-MSCs can improve kidney function in CKD models after infusion. da Silva et al. [51] injected the cultivated syngeneic BM-MSCs and their conditioned medium (CM) to a unilateral ureteral obstruction (UUO) rat through the abdominal vena cava. They found that both MSCs and their CM could decrease the expression levels of collagen 1, *α*-SMA, TNF-*α*, caspase 3, and proliferating cell nuclear antigen (PCNA) and ultimately reduce the EMT. However, they did not identify the detailed mechanism. In another study, renal-arterially delivered BM-MSCs can also reduce the EMT and renal fibrosis in rat UUO [52]. Wu et al. [53] injected BM-MSC uninephrectomized mice with bovine serum albumin- (BSA-) induced proteinuria to serve as CKD models. They determined that the BM-MSC-treated mice exhibited significantly reduced blood urea nitrogen (BUN) and urine albumin : creatinine ratio (UACR); moreover, collagen IV and *α*-SMA message RNAs (mRNAs) were prominently constrained with BM-MSC treatment. In their in vitro experiment, human proximal tubular epithelial cells (PTECs) cultured in albumin were obtained to mimic the pathological environment of CKD. They showed that EMT, *α*-SMA, fibronectin, and collagen IV mRNAs were significantly decreased with the presence of BM-MSCs. They also determined that this process was strongly associated with the phosphorylation of p38 and I-*κ*B, two transcript factors in the NF-*κ*B signaling pathway. In a transgenic mouse model that simulates human Alport disease, primary mouse BM-MSCs were injected into transgenic mice that are devoid of the a3-chain of type IV collagen (COL4A3) via the tail vein [54]. Ninichuk et al. [55] determined that no MSCs differentiated into renal cells, and the renal function was not promoted, although MSCs were found to localize to kidneys after injection. However, transplanted MSCs hampered peritubular capillary loss and reduced the idiosyncrasy of renal fibrosis, namely, the numbers of *α*-SMA-positive interstitial myofibroblasts, interstitial volume, and interstitial collagen deposition. Lang and Dai [56] performed a syngeneic BM-MSC transplantation in a streptozotocin- (STZ-) induced diabetic nephropathy rat model. They showed that the expression levels of TGF-*β*1, PAI-1, and Smad3 were reduced after MSC transfusion, which implies that the mechanism likely has a strong connection with suppressing the TGF-*β*1/Smad3 pathway. And this mechanism is also related to diminishing the expression of PAI-1 protein and abating the deposition of ECM, thus modulating the fibrinolytic system.

Additionally, human BM-MSC transplantation was popularly applied in animal models. Matsui et al. [57] used human BM-MSCs for rat UUO models through the renal artery. On the one hand, they showed that renal obstruction resulted in a substantial increase in the collagen, fibronectin, *α*-SMA, active STAT3, matrix metalloproteinase-9 (MMP-9), and matrix metalloproteinase-1 (TIMP-1, the tissue inhibitor) expression. On the other hand, exogenously administered human BM-MSCs could alleviate this obstruction-induced kidney fibrosis. And this could be done by inhibiting the activation of STAT3 and the production of STAT3-dependent MMP-9; these findings indicated that the JAK-STAT signaling pathway was involved in the mechanism of MSCs preventing renal fibrosis. Interestingly, MMP-9 has typically been considered an antifibrotic index due to its proteolytic activity [58]; however, recent studies indicate that MMPs, particularly MMP-9, are able to degrade the tubular basement membrane and shed away E-cadherin, thereby enhancing the EMT and fibrotic progression in obstruction-induced renal injury [46, 59]. In an Adriamycin- (ADR-) induced nephropathy mouse model, Song and his colleagues [45] showed that the levels of oxidative stress-related molecules (O_2_^−^, H_2_O_2_, malondialdehyde, and 4-HNE), proinflammatory cytokines (INF-*γ*, TNF-*α*, IL-12, IL-4, and COX2), NF-*κ*B, and ERK were significantly decreased with human BM-MSC administration. They also demonstrated these changes in an MSC-renal tubular epithelial cell cocultured system. Furthermore, BM-MSCs may use their secreted vesicles, such as exosomes, which may be loaded with useful nucleic acids and proteins to present to adjacent cells or targeted cells [60]. Using genetic engineering, Wang et al. [61] worked out microRNA- (miR-) let7c overexpressed in human BM-MSCs (defined as miR-let7c-MSCs), which exhibits a therapeutic capability in repairing or reversing established renal fibrosis. They found that miR-let7c-MSC therapy ameliorated kidney injury and markedly inhibited the expression of collagen IV, TGF-*β*1, MMP-9, and TGF-*β* type 1 receptor (TGF-*β*R1) in UUO mice. Furthermore, they utilized bioluminescence imaging and an exosomal inhibitor to visualize how miR-let7c-MSCs transfer miR-let7c by secreted exosome uptake. They also observed that after adding isolated exosomes or indirectly coculturing miR-let7c-MSCs with neighboring rat kidney tubular epithelial cells (NRK52E), the increasing expression of fibrotic genes induced by TGF-*β*1 in NRK52E was significantly repressed.

In summary, we showed that the vascular administration of BM-MSCs, as the most collected and earliest identified MSCs, or their subsidiary products (exosome or CM) could attenuate the progress of fibrosis and temper factors involved in inflammation, apoptosis, and the EMT. These factors typically involved several signaling pathways, mainly TGF-*β*1/Smad, as well as NK-*κ*B, JAK-STAT, and ERK. We summarized the details of every study above in Table 1 to make a clear display.

### 3.2. Umbilical Cord-Derived MSCs

UC-MSCs are overwhelmingly resourceful, and the collection process is safe and noninvasive [62]. Furthermore, UC-MSCs have presented a lower immunogenicity but a more effectively proliferative manner than BM-MSCs [63, 64]. Moreover, an increasing number of studies have confirmed that UC-MSCs exhibit promising therapeutic characteristics as an uprising resource of stem cells [65]. The umbilical cord is a conduit between the placenta and the developing embryo. During the prenatal phase, the umbilical cord is genetically and physiologically part of the fetus and usually contains two arteries (the umbilical artery) and one vein (the umbilical vein). All these blood vessels are buried in Wharton's jelly (WJ), which derives from the extraembryonic mesoderm and exerts a protective function [66]. Furthermore, WJ is considered a promising site for collecting MSC [67].

Hence, WJ-derived MSCs (WJ-MSCs) were studied specifically for their functions in treating renal fibrosis. Extraction of WJ-MSCs may be performed in the perivascular, intervascular, and subamniotic areas of the WJ [68]. Rodrigues et al. [47] set up a rat ischemia-reperfusion injury (IRI) model which received human WJ-MSCs intraperitoneally. They found that WJ-MSC-treated rats had better tubular function and promoted glomerular filtration, as well as lower levels of TGF-*β*1, macrophage infiltration, and the senescence-related protein *β*-galactosidase. Less macrophage infiltration might be equivalent to less severity of fibrosis [69]. Thus, rats treated with WJ-MSCs have a lower level of renal TGF-*β*1 and might present less chronic kidney impairment and fibrotic progression. Du et al. [49] labeled and injected human WJ-MSCs into unilateral IRI rats via the tail vein. No observation indicated that labeled cells resided in the impaired kidney. IRI-induced renal fibrosis was abolished by the treatment of these cells with the phenomena of a downregulated collagen content and *α*-SMA level. Moreover, the Akt signaling pathway was involved in these processes. To further investigate the mechanisms, the same team used the same method to establish an AKI rat model, and they identified one of the vital mechanisms: human WJ-MSCs could delay tubular EMT and ameliorate kidney fibrosis. The induction of foreign and native hepatocyte growth factor (HGF) biochemical synthesis at the primary stage of AKI in impaired tubular epithelial cells (TECs) contributes to the recovery of the imbalance between HGF/TGF-*β*1 during the renal scar-building process [70]. Moreover, Fan et al. [69] tested the effect of human WJ-MSCs in a rat model with peritoneal dialysis- (PD-) elicited fibrosis. And this model is established via PD solution with methylglyoxal (MGO) [71]. Intraperitoneal administration of human WJ-MSCs into rats considerably alleviated peritoneal fibrosis (decreasing the amount of collagen and *α*-SMA), the formation of the abdominal cocoon induced by PD/MGO, inflammation, neoangiogenesis, and ultrafiltration failure. They also proved there was a lower level of TGF-*β* in the WJ-MSC-treated groups, which indicated that the antifibrotic effect of WJ-MSCs might be related to the TGF-*β* signaling pathway.

Chinese scholars transplanted these icariin- (ICA-, extracted from a kidney-tonifying traditional Chinese medicine *Epimedium brevicornum Maxim* [72]) treated WJ-MSCs to an adenine-induced chronic renal failure (CRF) rat model via the tail vein. As a result, creatinine and BUN analyses reflected an improvement of renal function. IL-6 and TNF-*α* in the tissue homogenate and serum were substantially mitigated; however, the anti-inflammatory cytokine was increased. Moreover, the expression levels of bone morphogenetic protein-7 (BMP-7) and basic fibroblast growth factor (bFGF) were significantly higher [73]. In cultured renal cells, BMP-7 reacts against the profibrotic effects of TGF-*β* and exerts a protective function in both acute and chronic renal injury models [74]. Therefore, ICA-treated WJ-MSCs could reduce the levels of fibrosis in CRF rats. Moreover, ICA-treated WJ-MSCs were better than the application of WJ-MSCs alone as far as the antifibrotic and anti-inflammatory effects are concerned.

In addition, the functions of MVs derived from human WJ-MSCs have received substantial attention recently. Moreover, Zhang and his colleagues [75] injected MVs derived from human WJ-MSCs to the IRI rat, and they found that IRI-initiated fibrosis was abrogated by MVs coincident with renal function amelioration. This process was associated with the suppression of NADPH oxidase (NOX) 2 (the solitary function of reactive oxidative stress (ROS) production). In the same year, they showed that this antifibrotic process also had another potential mechanism. MVs derived from human WJ-MSCs could inhibit the chemokine CX3CL1 expression and reduce the CD68^+^ macrophage population in the kidney [76]. In the later stage, there were observations of renal function improvement and renal fibrosis amelioration in the MV-treated group. Furthermore, Chen et al. [77] recently added human WJ-MSC-MVs to NRK52E cells, and they found that these MVs could release from G2/M cell cycle arrest via the ERK1/2 signaling pathway. Therefore, these MVs could decrease the expression of collagen deposition-related proteins in NRK52E cells. In a rat donation after cardiac death (DCD) renal transplantation model established by Wu et al. [78], WJ-MSC-derived MVs were intravenously injected into rats immediately after renal transplantation. There were clear improvements in the renal function and survival rate. In the MV group, abolishment of renal fibrosis was identified in the late stage. Furthermore, MVs increased the HGF expression and decreased the *α*-SMA and TGF-*β*1 expression in all stages.

There were also some literatures which did introduce whether the UC-MSC came from WJ or not, so we defined these cells as unconfirmed UC-MSCs (uUC-MSCs). Huang et al. [79] injected uUC-MSCs into the UUO rat. The streptavidin-biotin complex (SABC) method was harnessed to track these uUC-MSCs after injection. Consequently, they found that these labeled uUC-MSCs were programmed to engraft an impaired kidney, particularly with the damaged renal interstitium. Also in the UUO model, Liu and his colleagues [80] collected the supernatants (CM) of uUC-MSCs and injected them via the renal artery. They found that this CM could significantly reduce the expression of EMT markers, such as *α*-SMA, TGF-*β*1, collagen I, and TNF-*α*. Moreover, the increasing level of E-cadherin reflected the prominent improvement of interstitial kidney fibrosis. The same team later performed similar experiments and identified a more detailed mechanism. In both UUO-induced in vivo and TGF-*β*1-induced in vitro experiments, human uUC-MSC CM could facilitate the renal function of UUO-induced rats and attenuate ECM accumulation, inflammatory cell infiltration, and their inflammatory cytokine release. Not only that, the uUC-MSC CM could also prominently inhibit the activation of TLR4/NF-*κ*B and the degradation of NF-*κ*B*α* both in vivo and ex vivo [48]. Moreover, Park et al. established a rat diabetic nephropathy model via an intraperitoneal injection of streptozotocin (STZ). Intravenously administered human uUC-MSCs efficiently decreased renal fibronectin, proteinuria, the downregulation of renal E-cadherin, and *α*-SMA upregulation in diabetic rats [81]. This study differed from other studies because a delayed treatment of uUC-MSCs was performed (4 weeks after diabetic nephropathy progression).

As previously described, human UC-MSC CM could significantly attenuate renal fibrosis [48, 80]. Some scientists address the true helpers of CM. Similar to BM-MSCs, the “cell-free therapy” strategy, the cell-derived extracellular vesicle (EV) for tissue repair has been broadly used on the basis of the paracrine or endocrine mechanism of MSCs [82]. Zou et al. [83] isolated EVs from the medium of human uUC-MSCs and injected them in rats intravenously after unilateral kidney ischemia. Moreover, the EV treatment could also improve the renal function and mitigate the histological lesion. Furthermore, an increase in the capillary vessel density and a reduction in the indicators of renal fibrosis (*α*-SMA and EMT) were observed after 2 weeks. EVs, according to the definition, are derived from cell membranes and are heterogeneous groups of vesicles that may be divided into apoptotic bodies (1–5 *μ*m), microvesicles (MVs) (100–1,000 nm), and exosomes (40–100 nm) according to their density, size, and biogenesis [84]. The diameters of the EVs in Zou et al.'s study ranged from 150 to 300 nm, which could be categorized as MVs [76]. Zhou and his colleagues [85] paid more attention to the smaller EVs, the exosomes. They used cisplatin (an anticancer drug) to induce acute kidney injury (AKI) rat models, and human uUC-MSC exosomes were injected into the renal capsule and ultimately into the injured kidneys. They found that there was a significant reduction in the creatinine levels and peripheral BUN, necrosis of proximal kidney tubules, apoptosis, and oxidative stress in rats, as well as the formation of a large amount of tubular protein casts after uUC-MSC exosome treatment. In vitro, the treatment of NRK52E cells with cisplatin alone resulted in a higher apoptosis level, activation of the p38 mitogen-activated protein kinase (p38MAPK) pathway, and increasing oxidative stress, followed by a decrease in cell multiplication and an increase in the expression of caspase 3; however, these processes were somehow alleviated and reversed in the uUC-MSC exosome-treated group. In addition, by activating the ERK 1/2 pathway, it was discovered that uUC-MSC exosomes enhanced cell proliferation. AKI, induced by IRI, contributes to kidney interstitial fibrosis, which is a critical characteristic of CKD and is therefore a high-risk factor in the progression of CKD [86]. Thus, the mechanism and process that occurred in Zhou's study could be strongly associated with renal fibrosis, and the MAPK p38 and ERK pathways also participated in the formation of renal fibrosis [40].

In addition, in ADR-induced nephropathic athymic rats, the therapeutic effect of transplanting three stromal cell populations (human BM-MSCs, uUC-MSCs, and kidney perivascular cells (kPSCs)) to engraft the damaged kidney via tail vein injection was evaluated. Rota et al. [31] found that all three cell populations protected podocytes from further loss, ameliorated glomerular endothelial cell injury, and weakened the production of PEC bridges and podocytes. This may be interpreted as a mitigation of fibrosis and glomerulosclerosis. By inducing polarization towards the M2 macrophage phenotype and reducing macrophage infiltration, human uUC-MSCs had the most preeminent anti-inflammatory effect compared to other stromal cells. CM from uUC-MSCs shared the same renoprotective properties. Similar to BM-MSCs, we made Table 2 to summarize the key information of every study above.

### 3.3. Adipose-Derived MSCs

Zuk et al. first successfully isolated MSCs from adipose tissue in 2001 [87]. Adipose tissue has since received considerable attention as an ideal MSC source due to its availability, abundance, and self-filling properties [88]. In vitro, cultured human AMSCs can grow adherently with fibroblast-like morphology [13]. Studies regarding AMSCs are very abundant, including autologous, syngeneic, allogeneic, and xenogeneic AMSC transplantation. The species include human, swine, cat, rat, and mouse.

In syngeneic and autologous AMSC transplantation studies, Song et al. [50] used AMSCs derived from the fat tissue that surrounded the epididymis of Wistar rats and transplanted these AMSCs into the UUO rat model through the tail vein. They observed that AMSCs substantially relieved kidney interstitial fibrosis by reducing the levels of fibroblast-specific protein 1 (FSP-1), *α*-SMA, EMT, and fibronectin, as well as the inflammatory response (release of IL-1*β*, TNF-*α*, and IL-6). They also investigated the following mechanism: AMSCs could inhibit TGF-*β*1/Smad2/3/7 signaling pathway activation. Burgos-Silva et al. [89] compared the efficacy of AKI and chronic kidney injury (CKI) via AMSC administration. Cultured FVB mouse AMSCs were intraperitoneally administered into FVB mice. As a result, the AMSC-treated animals showed an improvement in kidney function, and the chemokine expression (CXCL-1 and macrophage inflammatory protein 1 alpha (MIP-1*α*)) and neutrophil infiltrate were both reduced by AMSC administration. For CKI, renal fibrosis was ameliorated by decreased tissue chemokines, interstitial collagen deposition, and cytokine expression (INF-*γ* and eotaxin levels) in the AMSC-treated groups. Similar results were obtained in a mouse IRI model by Donizetti-Oliveira et al. [90], in which after animals showed established fibrosis at 6 weeks, syngeneic AMSC treatment manifested the promotion of renal function, accompanied by lower mRNA expression of collagen I and vimentin and reduced tissue fibrosis. Zou et al. [91] coated mouse AMSCs with antibodies directed against kidney injury molecule-1 (KIM1, a transmembrane protein that is expressed minimally in normal kidneys but upregulated dramatically in damaged kidneys [92]). The coating antibody ab-KIM1 has little influence on AMSC proliferation or viability. These AMSC-treated mice demonstrated an improvement in the capillary density and renal perfusion and attenuation of apoptosis, oxidative damage, and fibrosis (downregulated PAI-1, but no effect on TGF-*β* expression) in a unilateral renal artery stenosis (RAS) model. In a DCD kidney transplantation rat model, syngeneic AMSCs were injected in two different ways: systemic injection through the penile vein and local injection prior to angiostomy through the renal artery from the donor's kidney (local injection). Iwai et al. [93] discovered that local injection of AMSCs was prone to attenuation of fibrosis. In line with a literature, BM-MSC therapy may prevent long-term fibrotic changes in the allogeneic transplantation clinical setting [94]. This study suggested that the local injection of AMSCs was a potentially safer route of administration than a high-dosage, intravenous injection. Similar to BM-MSCs and UC-MSCs, there are also studies regarding the “cell-free therapy” of AMSCs; Eirin and his colleagues [95] ultracentrifuged EVs (both small MVs and exosomes) from pig autologous AMSCs and transplanted to the RAS pig model by laying a local irritant coil in the renal artery. Tubulointerstitial fibrosis (trichrome staining) was slightly but significantly decreased by AMSC-derived EVs.

For allogeneic AMSC transplantation studies, in 3 pilot studies, naturally occurring CKD cats enrolled in Quimby et al.'s studies [96] received allogeneic AMSCs every 2 weeks via intravenous injection; the AMSCs were obtained from donor-specific pathogen-free (SPF) cats' adipose tissue, and the extraction site was the same subcutaneous region on the ventral abdomen adjacent to the umbilicus. The authors reported that only one of the pilot studies showed a significant decrease in serum creatinine concentrations. Another similar cat study performed by the same research team indicated that there were no apparent alterations in BUN, serum creatinine, phosphorus, potassium, GFR by nuclear scintigraphy, UACR, or the packed cell volume in cats injected with allogeneic AMSCs [97].

For xenogeneic AMSC transplantation studies, Katsuno et al. [98] established human AMSCs cultured in low (2%) serum (hLAMSCs) and high (20%) serum (hHAMSCs) and performed a renal subcapsular injection of both hLAMSCs and hHAMSCs into a folic acid-induced AKI rat model. Interstitial fibrosis was less prominent in the hLAMSC-treated group. In contrast, in a cyclosporine- (CsA-) induced nephropathy rat model, after transfusion via the tail vein, human AMSCs did not prevent renal injury and interstitial fibrosis and manifested an inclination to further renal degradation. The mechanism might be that human AMSCs caused oxidative stress by the effect of CsA [99]. Moreover, exosome-related research is also involved in xenogeneic AMSC transplantation. Zhu and his colleagues [100] injected human AMSCs into a UUO rat model. They found that AMSCs could attenuate AKI and mitigate subsequent renal fibrosis (the levels of *α*-SMA, collagen I, and TGF-*β*1/Smad3 phosphorylation). However, these positive effects were demolished by a drug (GW4869) that inhibits the modulation release of exosomes from human AMSCs.  Table 3 shows the keynotes of all the studies above.

### 3.4. Amniotic Fluid and Placenta-Derived MSCs

To prevent diseases such as leukemia [101], lymphoma [102], and aplastic anemia [103], more and more umbilical cord tissues and cells are preserved following the birth of babies. However, other gestational tissues, including the amniotic fluid, placental membranes, and placenta, are constantly disposed as junk after birth. This junk contains abundant highly pluripotent stem cell resources, including MSCs. By conducting amniocentesis in the midterm, amniotic fluid-derived MSCs (AF-MSCs) may be obtained from a small amount of amniotic fluid [104]. The collection of amniotic fluid during routine cesarean deliveries is also a promising method [105]. A method of extracting MSCs from the placenta was introduced by Steigman and Fauza [106], who indicated that by chorionic villus sampling or blunt dissection, the fetal placental specimen could be mechanically separated from the maternal decidua [107]. Currently, almost all studies of AF-MSCs and placenta-derived MSCs (PMSCs) in renal fibrosis are in animal models.

Sedrakyan et al. [108] established a mouse model of Alport syndrome (Col4a5 knockout mice) and transplanted syngeneic AF-MSCs intracardiacally. The results showed that AF-MSCs prior to the onset of proteinuria delayed interstitial fibrosis and the progression of glomerular sclerosis. An M2 macrophage polarization was also identified in AF-MSC-treated mouse kidneys. Moreover, the fact that AF-MSCs suppress the renin-angiotensin system may contribute to these beneficial effects. Baulier et al. [109] used a porcine model to transplant autologous AF-MSCs directly into the grafted kidney via the renal artery 6 days after isogenic kidney transplantation. The injection of AF-MSCs enhanced tubular and glomerular functions, thereby abrogating fibrosis and increasing the levels of circulating TGF-*β*, which indicated that autologous AF-MSCs might develop their antifibrotic effect through the TGF-*β* pathway. Xenogeneic studies are about human AF-MSCs. In an IRI modeled rat, human AF-MSCs were transfused via the abdominal aorta. As a result, human AF-MSCs made significantly less inflammatory cell infiltration and tubular necrosis [110]. In a UUO mouse model, Sun et al. [111] showed that human AF-MSCs could increase the microvascular density, thus ameliorating interstitial fibrosis and protecting kidney functions.

Japanese scholars Tooi et al. [112] showed that human PMSCs confer plasticity on fibroblasts in vitro instead of directly in a fibrosis animal model. They found that PMSC exosomes considerably increased the expression of NANOG (stemness-related genes) mRNA and OCT4 expression in fibroblasts, and these exosomes influence the capability of fibroblasts to differentiate into both osteoblasts and adipocytes, which indicated novel characteristics of PMSCs and the promising potential of applying PMSC exosomes in clinical situations.

### 3.5. Renal Tissue-Resident MSCs

For one, MSCs could be derived from variant tissues, including the bone marrow, umbilical cord, and adipose tissue, and they have exhibited substantial promise in treating kidney fibrosis as previously described. For another, it has been shown that MSC-like cells in vivo reside in the pericyte or perivascular niche, and not only do they later become typical cultured MSCs but they also have strong connections with endothelial cells. Furthermore, they are much more likely to contribute to renal fibrosis by differentiating into myofibroblasts [113]. In the Hedgehog pathway, researchers have determined that Gli1 is a marker expressed specifically in a major population of tissue-resident MSCs, and these Gli1^+^ MSCs form a strong network across organs. Lineage tracing studies have shown that the Gli1 progeny covers injured areas rich in collagen I deposition [14], which indicates that these cells actively synthesize collagen and participate in ECM deposition during transient fibrogenesis.

By establishing UUO and IRI mouse models, Kramann et al. [23] tracked down the fate of genetically labeled Gli1^+^ MSC cells and determined that there was a substantial increase of Gli1^+^ cells throughout the kidney. The later ablation of Gli1^+^ cells after kidney injuries corroborated that there was an imposing amelioration of fibrosis and a stabilization of renal function in mice compared with the sham group. This discovery also applies to other organs, including the liver and lung [24]. Similar research was conducted by Humphreys et al. [114] by tracing the origin of myofibroblasts. After establishing a mouse UUO model, they discovered that kidney epithelial cells did not become myofibroblasts; in contrast, cells deriving from FoxD1, which is a transcription factor expressed in cells, and later becoming kidney mesenchymal stromal cells are the progenitor of myofibroblasts [25]. Studies performed by Leaf et al. [115] demonstrated additional mechanisms of kidney-resident MSCs leading to renal damages. They found that myeloid differentiation primary response 88 (MyD88) and its downstream effector kinase IL-1 receptor-associated kinase 4 (IRAK4) intrinsically control renal tissue-resident MSC migration and conversion to myofibroblasts. The reason is that IRAK4 and MyD88 regulate inflammation and fibrogenesis, which are the 2 major injury responses in the kidney [116].

### 3.6. Dental MSCs

Dental stem cells (DSCs) are MSC-like cells that exist in the human body throughout life [117]. There are five types of DSCs: stem cells from human exfoliated deciduous teeth (SHED), periodontal ligament stem cells (PDLSCs), dental pulp stem cells (DPSCs), stem cells from apical papilla (SCAP), and dental follicle precursor cells (DFPCs) [118]. Barros et al. [119] explored the homing of cryopreserved DPSCs in an acute renal failure (ARF) rat model. DPSCs demonstrated renotropic and pericyte-like properties, after intravenous or intraperitoneal injection, and contributed to accelerating the regeneration of the renal tubule structure. Furthermore, Hattori et al. [120] verified that in ischemia-reperfusion injury-initiated acute kidney injury, SHED was able to decrease inflammatory cytokines and promote kidney function. Yamaza et al. [121] investigated the immunomodulatory capacity of SHED as a potential treatment in a murine systemic lupus erythematosus (SLE) model. According to the results, the intravenous administration of SHED resulted in a significant reduction in the serum antibody levels, trabecular bone reconstruction, and regulation of Th17 cells. SLE and ARF were both proven to develop CKD (renal fibrosis) [122, 123].

### 3.7. Peripheral Blood MSCs

Among all tissues and cells, peripheral blood mononuclear cells (PBMCs) seem to be the most convenient to obtain. In research conducted by Pan et al. [124], human PBMCs were collected, purified, and reprogrammed by oocyte-inducing agents into induced mesenchymal stem cells (iMSCs). The iMSCs were subsequently injected into rabbit UUO models. The team indicated that significantly lower BUN and serum creatinine values and higher GFR as well as lower TGF-*β*1 and almost no fibrosis were identified in the induced group; this study suggested that iMSCs derived from human PBMCs could be a new shortcut in the treatment of chronic kidney diseases.

## 4. Attempts for MSC-Based Therapy in Clinical Application

As previously described, in most preclinical trials, MSCs exhibit few or no adverse side effects and are safe, while the efficacy remains controversial [96, 97, 109]. However, regarding translation to humans, safety concerns present a strong impediment to the smooth transition from laboratorial MSCs to pragmatic clinical MSC treatment. These safety concerns include the susceptibility to infection due to their immunomodulatory capabilities, potential canceration from the active proliferation of MSCs, embolism of the cells, cellular acute or chronic immunogenicity, and cell culture reagent-induced zoonoses [125]. A meta-analysis of the randomized control trials (RCTs) did not indicate a connection between organ system complications, infection, acute infusion toxicity, malignancy, or death [126]. In renal fibrosis, scientists have continued attempts with MSC-based therapy in clinical application. Foremost, in phase 1 of the clinical trial conducted by Togel and Westenfelder [127], patients who endured high postoperative AKI risks after cardiac operations safely received allogeneic MSC treatment. In a case of a living-related kidney transplant study, bone marrow tissue of the kidney recipient was collected from the posterior iliac crest to extract autologous BM-MSCs one month before the transplant. Tan et al. [128] found that there was a reduced risk of opportunistic infection, lower possibility of acute rejection, and better predicted kidney function at the 1-year follow-up using autologous MSCs compared with those of anti-IL-2 receptor antibody induction therapy. However, the autologous MSC inoculum was not associated with unfavorable events, and it did not negatively impact kidney transplant survival. Consequently, Reinders and his colleagues [129] carried out a series of clinical trials on the effects and safety of autologous and allogeneic BM-MSCs in renal fibrotic processes. They initially expanded BM-MSCs procured from end-stage renal disease (ESRD) patients and age-matched healthy controls who shared similar phenotypical and functional characteristics. Moreover, they performed a phase I study of autologous BM-MSCs on allograft rejection after renal transplantation. They found that there was tubulitis resolution in two patients of allograft rejection without tubular atrophy (IF/TA) and interstitial fibrosis. They proved that autologous BM-MSC therapy in transplant with IF/TA and subclinical rejection is practical and safe clinically [130]. They subsequently evaluated whether the combination treatment of autologous BM-MSCs with everolimus, the mTOR inhibitor, which enhances the withdrawal of tacrolimus, could reduce renal fibrosis in renal transplant recipients. They obtained a favorable result from MSCs in regard to renal fibrosis prevention, safety, and the preservation of renal structure and function, which would imply auspicious advancement for patients who are on kidney transplant waiting lists [131]. Out of worry of an antidonor immune response of allogeneic source cell therapy, one year later, they tried to use allogeneic BM-MSCs to confirm their effects and safety also for renal transplant recipients. They concluded that allogeneic MSCs were an effective and safe treatment alternative for renal transplant recipients, particularly when faced with indications where autologous MSC treatment is not technically possible [132]. Recently, a clinical trial from Iran revealed the feasibility and safety of autologous AMSCs in peritoneal dialysis patients (IRCT2015052415841N2). As a result, no unfavorable lesions and no catheter-related complications were found in the participants, and one patient developed an episode of peritonitis and another patient experienced exit site infection, which did not appear to be related to the procedure [133]. Of course, there was also an individual report which revealed potential nephrotoxicity after CKD patients received autologous MSC treatment. Kim et al. reported a case report about autologous AMSC administration for a patient with CKD. Before the MSC administration, the kidney function of the patient was consistently stable for years. Without any other implications of AKI, preexisting renal failure promptly invaded the kidney one week after the treatment of autologous MSC. Hemodialysis was applied 3 months after MSC therapy. Renal biopsy during dialysis demonstrated that with a few cells expressing CD117 and CD34, inflammatory cell infiltration and severe interstitial fibrosis could be observed [134]. Additionally, the researching scale has expanded to more than 500 registered clinical trials (http://clinicaltrials.gov/) in harnessing MSCs for therapeutic purposes at the current stage. We summarized the information of 13 registered MSC clinical trials for renal fibrosis and CKD in Table 4. Generally, the bench-to-bedside transition of MSCs in curing renal fibrosis is tantalizingly promising.

As far as dose and frequency are concerned, there is no exact guideline as to how many cells and how often should a patient be receiving MSC treatment. It is reported that 1 − 2 × 10^6^/kg of MSCs is most appropriate for the human body [135]. Some authors suggested that a patient should receive two doses of therapy with each time and 7 days apart between two therapies [129, 130]. Others only gave a range on the quantities of MSCs during the treatment; they believe that the frequency should be further elucidated, because various studies have shown that a single infusion may be protective in kidney diseases while two or more infusions have better effects [135].

## 5. Conclusion, Questions, and Perspectives

**Figure 3:**
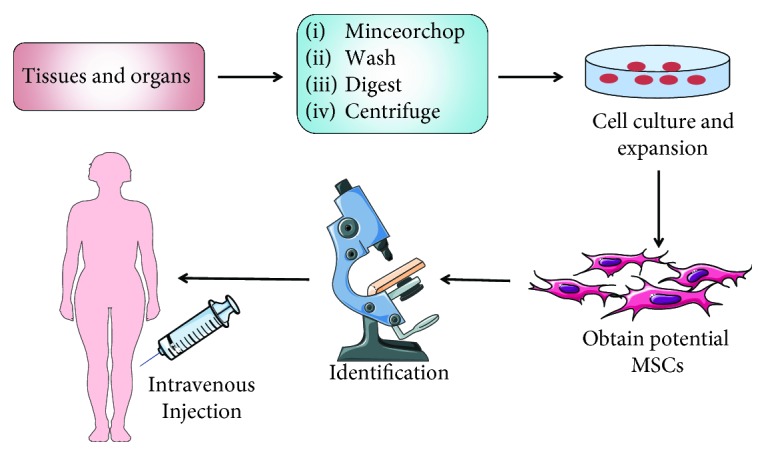
An overview of isolation and expansion procedures of MSCs from different tissues and organs and their administration in clinical application.

MSCs are found in almost all postnatal organs and tissues, including the fat, bone, cartilage, umbilical cord, cord blood, synovium, synovial fluid, muscle, skin, and pulp. MSCs from different sources possess similar morphologies and express identical surface markers. With their ability to differentiate into multiple lineages and migrate toward injured tissue and their propensity to secrete factors identified to be important in tissue recovery, MSCs undoubtedly have great therapeutic potential in renal fibrosis-related diseases. Preclinical and current clinical results seem promising, but moving the concept of MSC-based therapy forward to large-scale clinical application should be assessed critically. There are many ideas on how to manipulate the isolation and purification of MSCs to make them safer and more useful. Like many other cells, manipulation of MSCs in vitro and infusion of MSCs into CKD patients as a form of cell therapy will require many explorations before the real application in clinical practice (Figure 3).

Additionally, some controversial questions should be addressed when actually doing MSC-based therapy.

*(1) The Injection Way of MSCs*. Most injection ways of the studies discussed in this review are systemically intravenous (e.g., peripheral veins) and via the local artery (e.g., renal artery). The former one is more feasible in clinical operation, but the latter one is considered to be more effective because all the MSCs injected seem to localize in the kidney. In actual, there was no difference in therapeutic effects between the systemic and local ways of injection. Not in a kidney disease model, but in a model of permanent middle cerebral artery occlusion, Gutierrez-Fernandez et al. [136] proved that both MSC administration routes (external carotid artery and femoral vein) showed no difference in improving neurological recovery and provided brain protection. Together with the easy operation and low hurt, the systemically intravenous way is more recommended.

*(2) The Choice of MSC Heterogeneity*. Although xenogeneic human MSC transplantation is very common in animal models, the reverse way (animal MSCs to human) is mostly not considered currently because of safety. Therefore, choosing autologous or allogeneic MSC transplantation should be discussed in clinical application. Autologous MSC transplantation should be considered as the best way for MSC-based therapy because of the low immunogenicity and noninfectious feature. However, it takes several weeks to months to manufacture autologous cells due to the expansion period, quality controls, and logistics, and this period of time is too long for patients in need of treatment. Moreover, MSCs derived from patients with renal disease have a lower capacity for kidney regeneration [137]. Allogeneic MSCs offer an “off the shelf” advantage for clinical use and have the potential to be mass-produced rapidly [138]. This would significantly decrease costs and the number of procedures and enable use of cells from young healthy donors that may exhibit higher efficacy than cells from older individuals.

Last but not the least, many popular and novel techniques can be used for investigating the mechanism and clinical efficacy of MSC-based therapy in renal fibrosis.

*(1) RNA Array and Proteomics*. Because of the safety of cell therapy, delivery of MSC-derived EVs could be an attractive cell-free therapy for renal disease. Just like a pickup truck, EVs, as the delivery of cell-cell communication, can take many useful substances, mainly RNAs and proteins, to affect target cells [61]. Characterization of the transcriptome of human BM-MSCs and their relative-derived EVs using real-time quantitative polymerase chain reaction (RT-qPCR) arrays revealed that EVs contain a wide range of mRNAs [139]. Moreover, the study of miRNAs (thought to be the product of “junk DNA” before) is a rapidly expanding field, and in recent years, several miRNA families have been implicated in kidney fibrosis. According to RNA array, there are numerous miRNAs found in kidney disease, and the main families are miRNA-let-7, miRNA-21, and miRNA-29 [140]. Haraszti et al. [141] identified 3,532 proteins and 1,961 lipid species in MSC-derived EVs by high-resolution lipidomic and proteomic analyses. All the array techniques could provide abundant information for the mechanism of MSC-based therapy.

*(2) New Biomaterials*. Bae et al. [142] made a three-dimensional bioprinting of an artificial trachea carrying in vitro cultured rabbit BM-MSCs and respiratory epithelial cells. In addition, in order to solve the problems of the cell washout and immune attack during MSC administration, Hwang et al. [143] created an “intravascular paracrine factor (PF) factory” by harnessing stem cells on a stent using a nanofiber (NF) stent sleeve, thus providing a sheltered milieu for cells to continuously produce PFs on-stent. NF stent sleeves were created by covering stents with electrospun poly-lactic-co-glycolic acid nanofibers and were then uniformly coated with MSCs. Moreover, Huang et al. [144] found that exosomes derived from DPSCs and BM-MSCs could bind to the ECM, and they were deduced along with cell-derived ECM-incorporated biomaterials. And these exosomes can be used to recreate a complete extracellular environment that can enable safe and reliable differentiation of stem cells.

In summary, MSC-based therapy in renal fibrosis has become more and more acceptable because of their many advantages like ubiquitous sources, convenient procurement and collection, low immunogenicity, and low adverse effects. We believe it will keep the flame of cellular therapy burning and sparkling.

## Figures and Tables

**Table 1:** Literature summary of BM-MSC in the antifibrotic process.

Researcher	Fibrogenic signal pathway involved	Delivery method	Animal model	Heterogeneity
da Silva et al. [51]	Not mentioned	Abdominal vena cava	Rat UUO	Syngeneic
Asanuma et al. [52]	Not mentioned	Renal artery	Rat UUO	Xenogeneic (from human)
Wu et al. [53]	P38 and NF-*κ*B	i.v.	Mouse BSA-induced proteinuria (CKD model)	Murine
Ninichuk et al. [55]	Not mentioned	Tail vein	COL4A3-deficient mouse (Alport disease model)	Murine
Lang and Dai [56]	TGF-*β*1/Smad3	i.p.	Rat STZ-induced diabetic nephropathy	Syngeneic
Matsui et al. [57]	JAK-STAT3	Renal artery	Rat UUO	Xenogeneic (from human)
Song et al. [45]	NF-*κ*B and ERK	Tail vein	ADR-induced nephropathy mouse model	Xenogeneic (from human)
Wang et al. [61]	TGF-*β*1	i.v.	Mouse UUO	Xenogeneic (from human)

i.v.: intravenously; i.p.: intraperitoneally.

**Table 2:** Literature summary of UC- (WJ-) MSC in the antifibrotic process.

Researcher	Cell/substance transferred	Fibrogenic signal pathway involved	Delivery method	Animal model	Heterogeneity
Rodrigues et al. [47]	WJ-MSC	TGF-*β*1	i.p.	Rat IRI	Xenogeneic (from human)
Du et al. [70]	WJ-MSC	Akt and TGF-*β*1	Tail vein	Rat IRI	Xenogeneic (from human)
Fan et al. [69]	WJ-MSC	TGF-*β*	i.p.	Rat peritoneal dialysis- (PD-) elicited fibrosis model	Xenogeneic (from human)
Zhang et al. [62]	WJ-MSC MV	NOX 2	Caudal vein	Rat IRI	Xenogeneic (from human)
Wu et al. [78]	WJ-MSC MV	TGF-*β*1	i.v.	Rat DCD renal transplantation	Xenogeneic (from human)
Li et al. [73]	WJ-MSC	TGF-*β*	Tail vein	Adenine-induced CRF rat model	Xenogeneic (from human)
Huang et al. [79]	uUC-MSC	Not mentioned	Tail vein	Rat UUO	Xenogeneic (from human)
Liu et al. [80]	uUC-MSC CM	TGF-*β*1	Renal artery	Rat UUO	Xenogeneic (from human)
Liu et al. [48]	uUC-MSC CM	TLR4/NF-*κ*B	Renal artery	Rat UUO	Xenogeneic (from human)
Park et al. [81]	uUC-MSC	Not mentioned	i.v.	Rat STZ-induced diabetic nephropathy	Xenogeneic (from human)
Zou et al. [76]	uUC-MSC EV	HIF-1*α*	Caudal vein	Rat IRI	Xenogeneic (from human)
Zhou et al. [85]	uUC-MSC exosome	p38MAPK and ERK 1/2	Renal capsule	Cisplatin-induced AKI rat model	Xenogeneic (from human)
Rota et al. [31]	uUC-MSC	Not mentioned	Tail vein	ADR-induced nephropathic athymic rat	Xenogeneic (from human)

**Table 3:** Literature summary of AMSC in the antifibrotic process.

Researcher	Fibrogenic signal pathway involved	Delivery method	Animal model	Heterogeneity	Antifibrotic effect
Song et al. [50]	TGF-*β*1/Smad2/3/7	Tail vein	Rat UUO	Syngeneic	Yes
Burgos-Silva et al. [89]	Not mentioned	i.p.	AKI and CKI mouse models	Syngeneic	Yes
Donizetti-Oliveira et al. [90]	TGF-*β*	i.p.	Mouse IRI	Syngeneic	Yes
Zou et al. [91]	PAI-1	Carotid artery	Unilateral RAS mouse model	Syngeneic	Yes
Iwai et al. [93]	Not mentioned	Renal artery	Rat DCD renal transplantation	Syngeneic	Yes
Eirin et al. [95]	Not mentioned	Renal artery	RAS pig model	Autologous	Yes
Quimby et al. [96]	Not mentioned	i.v.	Cat CKD model	Allogeneic	No
Katsuno et al. [98]	Not mentioned	Renal subcapsule	Folic acid-induced AKI rat model	Xenogeneic (from human)	Yes
Chung et al. [99]	Not mentioned	Tail vein	CsA-induced nephropathy rat model	Xenogeneic (from human)	No
Zhu et al. [100]	TGF-*β*1/Smad3	Tail vein	Mouse IRI	Xenogeneic (from human)	Yes

**Table 4:** Registered clinical trials of MSCs in renal fibrosis and CKD (ClinicalTrial.gov, updated September 2018).

Registered no.	Status	Title	Population (age: year-old)	Phase	Source of MSCs	Location	Start date
NCT00659620	Unknown	Mesenchymal stem cell transplantation in the treatment of chronic allograft nephropathy	20 (18-60)	1 and 2	Bone marrow	Fuzhou, China	May 2008
NCT00733876	Completed	Allogeneic multipotent stromal cell treatment for acute kidney injury following cardiac surgery	15 (>18)	1	Bone marrow	Murray, Salt lake city, United States	August 2008
NCT00734396	Completed	Mesenchymal stem cells and subclinical rejection	15 (18-65)	1 and 2	Bone marrow	Leiden, Netherlands	February 2009
NCT01840540	Completed	MSC for occlusive disease of the kidney	6 (40-80)	1	Adipose	Rochester, United States	April 2013
NCT01967186	Unknown	Intraportal or intramuscular site for islets in simultaneous islet and kidney transplantation	36 (18-65)	Not applicable	Bone marrow	The Nordic network for clinical islet transplantation	April 2007
NCT02057965	Recruiting	Mesenchymal stromal cell therapy in renal recipients	70 (18-75)	2	Bone marrow	Leiden, Netherlands	March 2014
NCT02166489	Completed	Mesenchymal stem cell transplantation in patients with chronic renal failure due to polycystic kidney disease	6 (18-60)	1	Bone marrow	Tehran, Iran	March 2014
NCT02195323	Completed	Autologous bone marrow-derived mesenchymal stromal cells (BM-MSCs) in patients with chronic kidney disease (CKD)	7 (25-60)	1	Bone marrow	Tehran, Iran	April 2014
NCT02266394	Recruiting	Hypoxia and inflammatory injury in human renovascular hypertension	42 (40-80)	1	Adipose	Birmingham, Rochester, Jackson, United States	October 2014
NCT02387151	Active, not recruiting	Allogeneic mesenchymal stromal cell therapy in renal transplant recipients	10 (18-75)	1	Bone marrow	Leiden, Netherlands	March 2015
NCT02565459	Recruiting	MSC and kidney transplant tolerance (phase A)	22 (18-75)	1	Bone marrow	Bergamo, Italy	September 2015
NCT02966717	Active, not recruiting	Rituximab combined with MSCs in the treatment of PNS (3-4 stages of CKD)	116 (18-65)	2	Not mentioned	Guangzhou, China	August 2016
NCT03321942	Recruiting	Treatment of chronic renal failure with adipose tissue-derived mesenchymal stem cells	100 (18-75)	Not applicable	Adipose	Xuzhou, China	January 2017
